# Development and Validation of Population Clusters for Integrating Health and Social Care: Protocol for a Mixed Methods Study in Multiple Long-Term Conditions (Cluster-Artificial Intelligence for Multiple Long-Term Conditions)

**DOI:** 10.2196/34405

**Published:** 2022-06-16

**Authors:** Hajira Dambha-Miller, Glenn Simpson, Ralph K Akyea, Hilda Hounkpatin, Leanne Morrison, Jon Gibson, Jonathan Stokes, Nazrul Islam, Adriane Chapman, Beth Stuart, Francesco Zaccardi, Zlatko Zlatev, Karen Jones, Paul Roderick, Michael Boniface, Miriam Santer, Andrew Farmer

**Affiliations:** 1 Primary Care Research Centre Southampton United Kingdom; 2 University of Nottingham Nottingham United Kingdom; 3 Division of Population Health, Health Services Research & Primary Care University of Manchester Manchester United Kingdom; 4 University of Oxford Oxford United Kingdom; 5 Electronic and Computer Science Centre for Health Technologies University of Southampton Southampton United Kingdom; 6 Diabetes Research Centre University of Leicester Leicester United Kingdom; 7 Centre for the Study of Health, Science and Environment University of Kent Kent United Kingdom; 8 Public Health University of Southampton Southampton United Kingdom; 9 Nuffield Department of Primary Care Health Sciences University of Oxford Oxford United Kingdom

**Keywords:** artificial intelligence, social care, multimorbidity, big data, protocol, mixed method, long-term health

## Abstract

**Background:**

Multiple long-term health conditions (multimorbidity) (MLTC-M) are increasingly prevalent and associated with high rates of morbidity, mortality, and health care expenditure. Strategies to address this have primarily focused on the biological aspects of disease, but MLTC-M also result from and are associated with additional psychosocial, economic, and environmental barriers. A shift toward more personalized, holistic, and integrated care could be effective. This could be made more efficient by identifying groups of populations based on their health and social needs. In turn, these will contribute to evidence-based solutions supporting delivery of interventions tailored to address the needs pertinent to each cluster. Evidence is needed on how to generate clusters based on health and social needs and quantify the impact of clusters on long-term health and costs.

**Objective:**

We intend to develop and validate population clusters that consider determinants of health and social care needs for people with MLTC-M using data-driven machine learning (ML) methods compared to expert-driven approaches within primary care national databases, followed by evaluation of cluster trajectories and their association with health outcomes and costs.

**Methods:**

The mixed methods program of work with parallel work streams include the following: (1) qualitative semistructured interview studies exploring patient, caregiver, and professional views on clinical and socioeconomic factors influencing experiences of living with or seeking care in MLTC-M; (2) modified Delphi with relevant stakeholders to generate variables on health and social (wider) determinants and to examine the feasibility of including these variables within existing primary care databases; and (3) cohort study with expert-driven segmentation, alongside data-driven algorithms. Outputs will be compared, clusters characterized, and trajectories over time examined to quantify associations with mortality, additional long-term conditions, worsening frailty, disease severity, and 10-year health and social care costs.

**Results:**

The study will commence in October 2021 and is expected to be completed by October 2023.

**Conclusions:**

By studying MLTC-M clusters, we will assess how more personalized care can be developed, how accurate costs can be provided, and how to better understand the personal and medical profiles and environment of individuals within each cluster. Integrated care that considers “whole persons” and their environment is essential in addressing the complex, diverse, and individual needs of people living with MLTC-M.

**International Registered Report Identifier (IRRID):**

PRR1-10.2196/34405

## Introduction

### Background

Multiple long-term health conditions (multimorbidity) (MLTC-M) have been defined in the 2018 Academy of Medical Sciences policy report [1] as “The coexistence of two or more chronic conditions, each one of which is either a physical noncommunicable disease of long duration, such as a cardiovascular disease or cancer; a mental health condition of long duration, such as a mood disorder or dementia; an infectious disease of long duration, such as HIV or hepatitis C.” Globally, 1 in 4 people have MLTC-M, although estimates vary [2,3]. Prevalence increases with age from 54% for those over 65 years to 83% for those over 85 years [3]. MLTC-M are associated with decreased quality of life for each additional LTC, worse mental health, reduced functional status, and more severe morbidity [4]. Mortality risk increases, with meta-analyses reporting hazard ratios of 1.73 (95%CI 1.41-2.13) and 2.72 (95%CI 1.81-4.08) with 2 or more and 3 or more LTC, respectively, compared to people without MLTC-M [5]. The economic burden consumes 70% of the National Health Service budget, 65% of hospital bed days, and 50% of general practice (GP) appointments [3]. Societal and economic impacts include a lower likelihood of full-time employment and a greater likelihood of receiving assistance for unemployment and housing needs [6].

These impacts emphasize the need for a deeper understanding of MLTC-M in relation to physical health, mental health, and social well-being. Integrated care may have the potential to address MLTC-M more effectively, although current evidence offers a mixed picture of the efficacy of integration in addressing the complex care needs of this cohort of patients. Previous MLTC-M research in the United Kingdom shows that integrated services in MLTC-M contributes to higher patient satisfaction [7], increased perceived quality of care, and increased or improved patient access; it may potentially contribute to lower costs, although evidence related to reductions in the cost of provision is inconsistent [8]. An umbrella review reported that integrated care had limited costs of care by reducing emergency admissions and the length of hospital stay along with increasing care in the patients’ own homes; however, some of these findings are based on limited evidence [9]. Integrating health and social needs also has the potential to address growing health inequalities, as MLTC-M are more prevalent in low socioeconomic groups and require earlier social care input [10]. This might include, for example, poverty alleviation through support with benefits, citizen's advice, housing, and literacy, alongside input to facilitate access to disability allowances.

Data sets comprising millions of patient records including measures of health and socioeconomic determinants alongside subsequent health and social needs over the life course of a patient with MLTC-M are increasingly available. This provides opportunities to advance the understanding of MLTC-M toward the delivery of truly person-centered and holistic care. At present, efforts to improve care focus on approaches that primarily address biological needs, rather than considering the impact of wider health and social determinants on individuals living with several conditions at the same time [11]. This is because MLTC-M can be determined by and lead to socioeconomic and psychosocial barriers to health [12]. For example, poor cognitive function impacts treatment adherence, finances, or housing, and physical limitations impair access to health, healthy food, or green spaces for physical activity. A shift toward holistic and integrated care, alongside a preventative approach to health and social needs [13] could be effective in reorienting health and social care inputs to manage the complications, consequences, and costs of MLTC-M.

Operationalizing holistic and integrated care is challenging due to the level of personalization required across the health and social care continuum. At an individual level, it is costly and difficult to implement. Clustering heterogeneous populations into relatively homogenous subgroups with similar health and socioeconomic determinants and needs and then tailoring appropriate interventions to each cluster could offer a pragmatic solution. Studies have demonstrated the potential of clustering for integrating health and social care using expert-driven segmentation [14]. These methods face challenges in combining volumes of disparate data such as that found across social and community services. Evidence on population clustering uses expert-driven approaches based on a priori criteria [14]. This is limited by uncertainty regarding the completeness of the included variables and the number of natural clusters. Our recent review [15] and work by others show that a priori methods are more commonly used to group populations by single diseases as well as sociodemographic and clinical characteristics, and that these are within limited sectors rather than across the health and social care continuum [16]. Although studies report the potential of expert clustering approaches for integration of care [14], these have faced challenges in processing volumes of unlinked data across services with poor distinction between clusters and trajectories over time [17]. Data-driven approaches could process large amounts of disparate information to generate homogenous clusters [16,18].

Data-driven approaches include unsupervised artificial intelligence (AI) algorithms, including metric learning or variational autoencoder frameworks. The feature selection and engineering process will initially be informed by expert- and patient-proposed variables, but deep ML can extend these using self-learning. Clusters generated by deep artificial neural networks (ANNs) are more likely to be homogenous and predict trajectories. For example, a study of 2449 participants in Taiwan combined medical and socioeconomic data to generate data-driven clusters that accurately predicted service usage and expenditure [19]. In the Netherlands, biopsychosocial needs of the elderly were combined from 25 data sets across health, welfare, and elderly associations to generate data-driven clusters that informed resource allocation and finances [20]. Another study from Singapore (N=146,999) collated data from health and social care services using ML to produce clusters that were sensitive to changes in health status and progression toward disability [6]. Validity was supported by the ability of clusters to discriminate between longitudinal health care usage and mortality. Global evidence suggests that data-driven clustering using AI has the potential for understanding MLTC-M and establishing care systems based on the principles of person-centered care. Such systems are expected to provide opportunities for better and more timely interventions, a reduction in disease burden, and better use of scarce resources, and these now need to be examined in the United Kingdom.

Advances in data-driven processing paradigms could overcome previous limitations in methodology using unsupervised or semisupervised deep embedded clustering [16,18,21]. ML can process rich longitudinal records to discover natural groupings of data points with or without knowledge from human experts in the form of ground truth labels or feature constraints. Traditional algorithmic approaches include k-means, Gaussian mixture models, hierarchical and Bayesian network–based clustering, whereas recent approaches use deep ANNs [22]. Such algorithms can provide cohesive groupings based on self-learned features and are more likely to predict trajectories toward disease progression, frailty, and mortality [23]. Detailed characterization and comparison of clusters by “whole person” parameters can be analyzed that are unbiased by human understanding and consider sociodemographic and clinical profiles, service usage patterns, critical time points for change in needs, and deviation in comorbid disease severity. Using a “whole person” approach could inform the development of an intervention that would support health and social care providers to address the needs pertinent to each MLTC-M cluster and potentially provides an opportunity for efficient implementation of person-centered care.

### Objectives

We aim to develop and validate population clusters that consider health and social care determinants and subsequent health and care needs for people with MLTC-M using data-driven AI methods compared to expert-driven approaches, followed by evaluation of cluster trajectories and their associations with health outcomes and costs.

## Methods

We will carry out a longitudinal mixed methods study with 3 parallel work streams including (1) qualitative interview study, (2) modified Delphi, and (3) cohort study. They are described below.

### Qualitative Interview Study

#### Recruitment and Sampling

Email and postal invitations will be sent to participants who have expressed interest through advertisements viewed on social media, local community centers, the university website, charity newsletters, caregiver support networks, and through word of mouth. Given the complex structure of health and social care, an iterative and a proactive recruitment approach will be necessary. To include hard-to-reach and underrepresented groups that reflect diversity in social needs, we will recruit at events, such as those held in local authority facilities and community or faith centers, as well as seek additional expert input through established Black and minority ethnic networks. We will aim for a representative sample of <30 interviews, as our pilot interview study [24] showed that this number is achievable within the study timelines and data saturation was achieved with a sufficiently diverse sample of stakeholders across a range of geographies. Purposive sampling will be employed to capture a wide range of participant perspectives from across a diverse range of settings, as well as snowball sampling from the initial round of 10 interviews to identify further participants.

#### Data Collection

Semistructured interviews will explore views on health and social needs over the course of living with or supporting MLTC-M and views on possible intervention components identified from our preliminary work. Telephone or internet-based video interviews will be conducted by trained researchers. An interview schedule will be designed covering broad open questions to enable similar topics to be addressed across the sample. The design of the interview schedule will be informed by our study aims, our previously published scoping review, and the expertise of team members; it will then be tested prior to use. Furthermore, the development of the interview schedule will be iterative; insights from earlier interviews may inform additions or amendments to the interview schedule in the later interviews. A flexible approach will be used to ensure that related subjects of importance can be raised. Interviews will be digitally recorded and transcribed verbatim and the content anonymized.

#### Analysis

We will use inductive reflexive thematic analysis [25,26]. Throughout the analytical process, a form of constant comparative analysis will be used to identify key differences or similarities in the data, between professions, and sectors or geographies. Discussion of the evolving analysis within the research team will enable us to explore and incorporate alternative perspectives that may challenge and enrich initial interpretations of the data. Additionally, we will use deviant case analysis to further broaden (or confirm) the patterns identified in the data, adding rigor to our analytical conclusions. A summary of our findings will then be sent to a sample of participants who had agreed to receive them to ensure that we have appropriately captured relevant points of view. QSR NVivo software (version 12) will be used to manage the data and the COREQ (Consolidated Criteria for Reporting Qualitative Research) checklist will guide reporting [27].

### Modified Delphi

#### Theoretical Framework

Integrated care with co-ordination, continuous health, and social input is set out by the SELFIE (Sustainable intEgrated care models for multi-morbidity: delivery, FInancing and performancE) framework [28]. This conceptual model considers patients with MLTC-M, emphasizing holistic understanding of individuals in their own environment at its core. The micro-, meso- and macroenvironments branch from this central point. By starting at the core and moving around the framework, researchers, policy makers, and practitioners are guided through considerations of individuals, their health, social context, and the wider environment.

#### Recruitment and Sampling

A “virtual Delphi panel” will be established. Participants will be invited to join, including experts from health and social care, service managers, researchers, caregivers, patients, and database managers. We will convene a panel of >20 members. A purposive sampling approach will be used to recruit the panel.

#### Method and Analysis

A modified Delphi technique [29] conducted over 3 rounds will be used to collect expert views on clinical and socioeconomic determinants, and subsequent needs over the life course of MLTC-M. Throughout, the process will use the SELFIE framework that conceptualizes integration of care at the micro, meso, and macro scales according to the 6 key (World Health Organization) components (service delivery, leadership and governance, workforce, financing, technologies and medical products, and information and research) [28]. This conceptual framework is presented diagrammatically in a layered pie chart model, with the individuals with multimorbidity and their environment placed at its core. Radiating out from the core, concepts pertaining to integrated care for multimorbidity are grouped at the micro, meso, and macro levels. They are further split according to the 6 key components of health systems used to describe, understand, and compare different global health systems.

Discussion among panelists related to the potential clustering of specific variables will be guided and structured by the SELFIE conceptual framework. In particular, the extent to which variables are applicable within existing databases or obtainable through new health and social care data linkages will be considered in detail by the panel. Initially, participants will be supplied with a ranked list of variables generated from our preliminary review, followed by discussion and the qualitative study described above. Then, similar ideas emerging from these discussions will be grouped. Potentially relevant variables will be collated by the research team, fed back, and subsequently rated or ranked by panelists at the next round (phase II), with a “free text” option available for clarification. The most highly rated variables will be taken forward (phase III).

The panelists in round 1 will make their initial judgments individually without any interaction with other panelists, and these “ratings” will be fed into subsequent rounds. Web-based interactions with other panelists will occur during the deliberation rounds of the Delphi panel, a process spanning 1 to 2 days. At each stage, researchers experienced in the Delphi method will moderate the panel. The research team will take notes of these discussions to track the decision-making process and determine how and why specific decisions are reached. No attempt will be made by researchers to hasten discussion or compel the panel to reach a consensus.

### Cohort Study

#### Data Sources

We will use the Clinical Practice Research Database (CPRD) GOLD and Aurum [30] to identify clusters. To test the validity of our approach and clusters identified, we will run our code on additional databases (eg, Secure Anonymised Information Linkage [SAIL]) and local data sets (eg, English Longitudinal Study of Ageing [ELSA]).

CPRD GOLD and Aurum include 50 million registered GP patients with high levels of heterogeneity in ethnicity, deprivation, and morbidities. Primary care–linked records include Hospital Episode Statistics Admitted Patient Care (HES APC) data on hospital admissions, discharges, accident and emergency (A and E), and outpatients in England, socioeconomic status (Index of Multiple Deprivation [IMD] or Townsend score), and death data from the Office for National Statistics.

SAIL is a nationwide repository of routinely collected electronic data on health and social care in Wales, United Kingdom. It includes over 2 billion anonymized records linked with hospital admissions and primary care data [17].

The ELSA collects data from people aged over 50 years covering physical and mental health, well-being, finances, and attitudes around aging and how these change over time. The Health Survey for England is an annual survey that looks at changes in the health and lifestyle of people. Local area data sets allow local authority data linkage with health information using health determinants from the census and social determinants, such as wealth and the IMD (a score calculated for each participant's neighborhood based on social indices such as income, education, and employment).

The same variable definitions will be applied to all data sets, wherever possible, to ensure consistency and comparability of findings from the respective data sets.

#### Population

Participants must be aged 18 years and over when entering the study. They must be diagnosed with MLT-C (defined by Guthrie et al, forthcoming) that included the following 59 conditions: stroke, coronary heart disease, heart failure, peripheral arterial disease, heart valve disorder, arrythmia, venous thromboembolic disease, aneurysm, hypertension, diabetes, Addison disease, cystic fibrosis, thyroid disease, chronic obstructive pulmonary disease, asthma, bronchiectasis, Parkinson disease, epilepsy, multiple sclerosis, paralysis, transient ischemic attack, peripheral neuropathy, chronic primary pain, solid organ cancer, hematological cancer, metastatic cancer, melanoma, benign cerebral tumors that can cause disability, dementia, schizophrenia, depression, anxiety, bipolar disorder, drug or alcohol misuse, eating disorder, autism, posttraumatic stress disorder, connective tissue disease, osteoarthritis, osteoporosis, gout, long-term musculoskeletal problems due to injury, chronic liver disease, inflammatory bowel disease, chronic pancreatic disease, peptic ulcer, chronic kidney disease, end-stage kidney disease, endometriosis, chronic urinary tract infection, anemia (including pernicious anemia, sickle cell anemia), visual impairment that cannot be corrected, hearing impairment that cannot be corrected, Meniere disease, HIV/AIDS, chronic Lyme disease, tuberculosis, postacute COVID-19, congenital disease, and chromosomal abnormality.

#### Follow-up

The participants within the data sets will be followed-up until the earliest occurrence of the following: developing the outcomes of interest, transfer out of the practice, death, practice stopping data contribution to the database, and end of data linkage to HES APC and the ONS.

### Clustering Variables

Variables for inclusion in the clustering models will be generated by the qualitative study and Delphi, including but not limited to sociodemographic variables (eg, age, sex, ethnicity, and IMD), clinical variables (eg, blood pressure, cholesterol, medication use defined as repeat medication only), social needs (eg, social services, physiotherapy, or occupation health input), health and social care usage (eg, hospitalization, outpatient appointments), mortality and health care costs (eg, inpatient costs of admissions to hospital as a day case [31] or as an inpatient for ≥1 night), outpatient and A and E costs, noninpatient costs (costs of all GP contacts and outpatient clinics) and for all other purposes, including primary care and social care, and Personal Social Services Research Unit costs [32].

### Outcome Variables

The initial core variables that we have included are as follows: all-cause and cause-specific mortality*,* deterioration to worsening MLTC-M (ie, number of conditions), worsening frailty score, inpatient costs (admission to hospital as a day case or as an inpatient for ≥1 night), outpatient costs, A and E cost, GP contract cost, social care contact cost, referral to community services (occupational therapy, district nurses, physiotherapy) cost, nursing home cost, and respite care costs. Additional outcome variables could be added depending on the results of the parallel qualitative and Delphi work package.

### Analysis

#### Traditional Statistical Modeling

Participant characteristics will be summarized with appropriate summary statistics. Using generalized linear models, we will describe multimorbidity rates and frailty over time. Survival models will be used to investigate all-cause and cause-specific mortality*.* For expert-driven clustering, we will carry out latent class modeling considering profiles over at least a 10-year follow-up period. We will model trajectories of multiple long-term conditions, as well as health and social care needs over time using group-based multitrajectory modeling in Stata (StataCorp). We will conduct complete case analyses for all models and separately evaluate the models in multiply imputed data (sensitivity analysis). Under the “missing at random assumption,” where appropriate, we will use multiple imputation with chained equations to generate 5 imputed data sets. Continuous values may need transformation before imputation. Imputation models will include all exposure and outcome variables; statistical models will be developed on each of the 5 imputed data sets and the estimates pooled using Rubin’s rules. Data will be analyzed using Stata (version 17). We will conduct our study and report the findings in line with the STROBE (Strengthening the Reporting of Observational Studies in Epidemiology) and RECORD (The REporting of studies Conducted using Observational Routinely collected health Data) guidelines for observational studies using routinely collected health data.

#### AI-Based Modeling

A variety of data mining and ML methods will be used for data-driven knowledge elicitation regarding the concept of social care needs, patient social care need trajectories over time, outcomes associated with the trajectories, and interventions (modifiable exposures) that can be used to modify the trajectories and the respective the final outcomes. This information will be included in a report on intervention strategies and policies.

The sequence of ML-based analytic tasks will be as follows:

1. The variables defining the concept of social care needs, which are generated by the modified Delphi study, will be entered for clustering and cluster interpretation to discover naturally occurring classes of social care needs. The clustering approach will exploit the ability of the ML methods to process high-dimensional and high-volume data using data science pipelines composed of dimensionality reduction, unsupervised clustering, supervised learning, and model interpretation algorithms. These pipelines are further described below.
2. Using longitudinal data, patients’ social care need trajectories (ie, sequences of social care need class membership) will be composed, and these trajectories will be clustered with respect to outcomes of interest, including mortality, worsening frailty, accrual of critical LTCs of interest, and costs. For trajectory clustering, we will apply hierarchical clustering with custom distance measures based on the outcomes of interest using the HDBSCAN (Hierarchical Density-Based Spatial Clustering of Applications with Noise) algorithm] in Python.
3. Trajectory clusters will be analyzed by experts for identifying clusters of interest (ie, clusters with undesirable outcomes). Further, intervention points will be identified aiming at trajectory modification so that trajectory outcomes can be positively modified.
4. Predictive and causal associations between exposures (these variables will be selected during the modified Delphi study) and trajectories will be modeled at the points for interventions previously selected. For predictive modeling, we will use the XGBoost algorithm in Python. For causal modeling, we will use directed acyclic graphs and linear models.

For the ML-based clustering in step 1 of the above procedure, we will develop, apply, and evaluate 2 ML clustering pipelines. Pipeline 1 is semiautomated based on shallow ML clustering using expert- crafted features calculated from raw data. Here, the expert is added by ML tools for data visualization by low-dimensional embedding. Pipeline 2 is a fully automated pipeline based on deep ANNs and explainable AI techniques, where the pipeline takes raw data as the input and provides clusters and interpretations as the output. In addition, prior to feeding data into the clustering pipelines, data will be preprocessed by rescaling of the numerical data (such as standardization or min-max scaling) or transformations for mixed categorical and numerical data (such as Gower transformation) or calculating dissimilarity measures for categorical data (such as the simple matching coefficient). The precise data preprocessing method will be finalized after a descriptive analysis of the selected variables.

Pipeline 1 uses semiautomated ML motivated mainly by the work of Becht et al [22] through applying Uniform Manifold Approximation and Projection (UMAP), t-Distributed Stochastic Neighborhood Embedding (t-SNE), and specialized variational autoencoder (SCVIS) algorithms based on biological cell type and cell differentiation trajectory clustering.

In phase 1, we interactively assess the propensity of the data for clustering and topology using low- dimensional (2D and 3D) embeddings with parameterized t-SNE and UMAP [22] and quantitative measures of goodness of clustering. If t-SNE and UMAP produce clusters, this would naturally mean that the input feature space contains the necessary information for separating the data into naturally occurring clusters and classes. If clusters cannot be observed in the low-dimensional space, new features derived by experts will need to be introduced. The t-SNE and UMAP algorithms expose information about the number of natural clusters in the data and the shape of the clusters, which will inform the selection of models to be used for fitting the clusters of data. In this role, it is generally reported that UMAP performs better when t-SNE [22] with a notable exception being nested cluster extraction (ie, a tight, dense cluster inside a wide, sparse cluster), where t-SNE performs better and UMAP fails to separate the clusters [33].

The data in the low-dimensional (2D/3D) output space of t-SNE and UMAP will be clustered using HDBSCAN; the quality of these clusters will be quantitatively evaluated using measures for cohesion and separation (eg, sum of squared errors, silhouette coefficient, Calinski-Harabasz and Davies Bouldin Indexes). The observed natural clusters in the low-dimensional (2D/3D) space are not explicitly and directly interpretable, as UMAP and t-SNE perform highly nonlinear transformations and further interpretable ML methods will be used in the next phase to facilitate their interpretation.

Phase 2 selects data features (<20) and interpretable ML classification algorithms to fit models onto the natural clusters in the data. Algorithms with interpretable models are, for example, decision trees, rule learners, naive Bayes, k-nearest neighbors, generalized linear models, Gaussian mixture models, or ensembles of these, each generally performing differently depending on the density, shape, and separation boundaries of the data classes. We will output interpretations of individual clusters based on the derived decision boundaries of the best performing classification model learned for the clusters.

Pipeline 2 is fully automated and builds on the approach by Ding et al [23] for applying deep generative models (autoencoders) to derive interpretable low-dimensional features for discovering biological cell types, states, and development lineages [23]. We will use this approach to discover homogenous patient groups and their health care–related states and trajectories. Pipeline 2 will be compute-intensive and use existing high-performance computing infrastructure including servers with graphic processing unit arrays.

In phase 1, automated dimensionality reduction will be performed with a deep learning autoencoder neural network, followed by clustering in the lower-dimension space, based on the work of Xie et al [34].

The autoencoder is given the raw data (even if it is very high-dimensional) and features are successively generated automatically by the layers of the autoencoder, as well as low-dimensional embedding for clustering. Importantly, the mapping of input features (the high-dimensional space) and output features (the low-dimensional space) is captured by the weights of the encoding and decoding neural networks of the autoencoder; we can make transformations between the 2 spaces, which are needed for deriving cluster interpretations using explainable AI techniques in phase 2. Further, in the low-dimensional space, automated clustering will be performed using HDBSCAN, where metrics for cohesion and separation (as in pipeline 1) will be used to select the best clustering.

In phase 2, explainable AI (XAI) algorithms will be used for deriving cluster interpretations. We will use the XGBoost algorithm coupled with the SHAP (SHapley Additive exPlanations) XAI algorithm [35] for building and interpreting models of the clusters derived in phase 1.

Beyond the approach and methods described above, and in the course of ongoing data analysis work, further refinement of our approach would be considered when addressing the specific concerns explained below.

### Causality

Statistical dependences between the studied variables will be inferred from the data, which will then be appraised by a multidisciplinary team for the type of relation (causal or not) and factored in dynamic epidemiological models. Interpretable ML will be used for determining dependences between variables, where the techniques will include Bayesian network inference from data, SHAP estimation, and local surrogate model inference (local interpretable model-agnostic explanations [LIME]) over learned and possibly nonlinear, models. Dictionary learning of sparse coding techniques will also be researched for selecting candidate causalities.

### Sparsity

This can naturally occur in high-dimensional spaces. However, some or many dimensions will not generally be mutually independent and there will be redundant dimensions to some degree with respect to a given learning task. This type of data sparsity will be addressed using dimensionality reduction techniques (eg, principal component analysis, UMAP) in the ML pipeline. Sparse data will also be interpreted by topological data analysis (eg, with the KeplerMapper tool) and manifold learning. For cases with sparse labeled data, but denser unlabeled data, semisupervised learning and the transfer learning pipeline will be specifically explored and designed for specific characteristics of the problem.

### Temporality

Time series and sequence learning for predicting the sequence of accrued conditions and cluster trajectory prediction will be performed with the long short-term memory (LSTM) and transformer ANN algorithms.

### Networked Data

We assume that some known relations between the studied variables exist and are machine readable. These relations will be used to create a graph representation of the related variables. The graph will be used with graph neural networks for learning better latent representations for the downstream learning tasks or for inferring unobserved relations.

### Explainability

Dimensionality reduction, visualizations, clustering, and topological data analysis will be used for finding naturally occurring structures in the data that can be related to the studied phenomenon. We will initially apply readily interpretable ML techniques such as linear models, decision trees, and inferred Bayesian networks. Furthermore, nonlinear model learning on the labeled data (supervised learning with XGBoost or deep ANN) and interpretation learning (with SHAP and LIME) will be performed for identifying predictor variables that will be further assessed for their epidemiological meaning by a multidisciplinary team.

### Bias

Addressing bias is critical to ensure model fairness and to ensure that predictions are not affected by an individual belonging to one of the groups defined by some sensitive attribute(s). An interdisciplinary approach is necessary to ensure that all researchers adopt principles of fairness and responsible AI practices [36,37]. Bias can be an intrinsic property of the data set resulting from sampling procedures and will be addressed qualitatively through engaging experts and data owners. Bias can also be introduced by decisions of data scientists during data preprocessing.

Data set selection, wrangling, and transformation have the potential to remove or inadvertently introduce new bias in data. We will address this challenge by building on our team’s existing work in this area including previous algorithms [36,37]. Our approach will apply a formal model of data preprocessing operations (feature selection, feature engineering, imputation and listwise deletion, resampling, outlier removal, smoothing/normalization, and encoding) to record the effect each operator has on the data. Operations will be logged during processing through code instrumentation to produce an end-to-end provenance document that contains a fine-grained history of each data set element in the final training set.

Derived clusters and interpretations from the expert-driven and AI-supported clustering will be summarized with descriptive statistics. This will also allow inequities in care across clusters to be assessed using a proxy of area-level deprivation for socioeconomic status. Clusters will also be analyzed to identify whether they differ statistically between the 2 methods. Appropriate regression models (depending on data distribution) will be constructed to quantify the association between population clusters and outcomes. Finally, we will compare outcomes between the derived population clusters with respect to key covariates and between the clustering methods.

We will conduct segmentation via latent class analysis using Latent GOLD (Statistical Innovations) and data-driven analysis will be performed with R (version 4 or later) and Python (version 3 or later). The Delphi panel will be reconvened for algorithmic stewardship that permits an additional layer of quality control to discuss AI outputs in terms of safety, fairness, effectiveness, and practicality and to determine which clusters to take forward [38].

We will ensure FAIR (Findable, Accessible, Interoperable, and Reusable) stewardship of data, curation of models, and research integrity through robust governance, as the pipeline develops processes building on the blueprint outlined for the Social Data Foundation, which has been written by one of the authors of this protocol [39]. Each AI pipeline will be developed using microservices deployed in containers (Kubernetes, Cloud Native Computing Foundation) and described using container orchestration for repeatable and continuous testing and deployment. Models will be developed in interactive environments such as Jupyter and built into software libraries for integration into automated workflows (eg, TensorFlow). Artifacts such as software libraries, images, and notebooks will be made available through open-source licenses allowing results to be replicated by others and research outputs repeated/compared by others.

Findable data will be indexed and annotated with semantically rich metadata using shared terminologies. Data will be made accessible by publishing them to national data services via application programming interfaces and directly by humans or machines for integration into workflows, whereas interoperable data will be aligned with standards such as Health Level Seven and Fast Healthcare Interoperability Resources. Reusable data require clear licensing including any ethical, legal, and security requirements necessary for usage.

### Ethical Approval

Ethical approval was granted from the University of Southampton Faculty of Medicine Research Committee (reference 67953).

## Results

This study is due to commence in October 2021 and we will aim to complete it by October 2023. The study received funding from the National Institute for Health Research.

Our research attempts to offer commissioners and policy makers reliable evidence on a new approach to manage MLTC-M. We will examine the potential of using ML methods to deliver insights into new disease clusters that consider health and social needs. Clusters will be profiled to evaluate differences in sociodemographic, clinical and treatment variables, comorbid disease patterns, and trajectories of disease progression. We will compare associations of disease trajectories with respect to outcomes and then conduct an intervention development phase to examine the feasibility of using advanced AI outputs to tailor the design of an intervention that supports the integration of care needs. This phase will develop the program theory and scope intervention content, as well as identify and address implementation, trust, adoption, and scalability issues to support rapid incorporation into existing service pathways. The generated evidence could provide a powerful tool for delivering holistic care and reducing the human cost and resource burden of MLTC-M.

## Discussion

### Principal Considerations

In this mixed methods program of work, we will use multiple large national primary care databases alongside qualitative work and a modified Delphi method to identify clusters of MLTC-M populations based on their health and social needs. Understanding these clusters and their trajectories over time will, in turn, help develop evidence-based solutions. These will be aimed at supporting the delivery of interventions tailored to address the needs pertinent to each homogenous cluster. Our evidence will provide key knowledge on how to generate clusters based on health and social needs and how to quantify the impact of clusters on long-term health and costs.

### Limitations

For our qualitative work, we will primarily be carrying out telephonic or virtual interviews whereas the modified Delphi method is an exclusively internet-based one. It is likely that respondents will include those able to use virtual technologies for interviews and those able to use and access virtual services to complete the web-based Delphi. People with MLTC-M who are unable to use these technologies, such as the elderly, those with disabilities, or those from lower socioeconomic backgrounds who may not have access to internet-based services, will not be sufficiently represented in our sample. It is plausible that the richer qualitative data could be obtained through in-person interviews.

There are inherent limitations associated with the analysis carried out on secondary data. These data have been collected in the clinical setting and are not for research purposes. They will have variations in entries and coding that are dependent on individual clinicians. Incomplete, missing, and incorrectly coded records are likely to be limitations. The extent to which problem exists in our data and the impact on our findings will require thorough exploration. Although our cohort will include primary care populations from several large national databases across large geographical areas in England and Wales, it may not include a sufficiently diverse sample of people from varying ethnic and socioeconomic status, thus limiting generalizability.

### Conclusions

Outputs from the research will offer commissioners and policy makers reliable evidence for a new approach to manage MLTC-M. Using a “whole person” approach could inform tailoring of interventions specific to each MLTC-M cluster. The evidence generated by this research has the potential to serve as a powerful tool for delivering holistic personalized care, thereby reducing the human cost and resource burden of MLTC-M.

## Abbreviations

A and E: accident and emergency
AI: artificial intelligence
ANN: artificial neural network
CPRD: Clinical Practice Research Database
ELSA: English Longitudinal Study of Ageing
GP: general practice
HDBSCAN: Hierarchical Density-Based Spatial Clustering of Applications with Noise
IMD: Index of Multiple Deprivation
LIME: local interpretable model-agnostic explanations
ML: machine learning
MLTC-M: multiple long-term health conditions (multimorbidity)
SAIL: Secure Anonymised Information Linkage
SELFIE: Sustainable intEgrated care models for multi-morbidity: delivery, FInancing and performancE)
SHAP: SHapley Additive exPlanations
t-SNE: t-Distributed Stochastic Neighborhood Embedding
UMAP: Uniform Manifold Approximation and Projection
XAI: explainable artificial intelligence
